# Fossil Plotopterid Seabirds from the Eo-Oligocene of the Olympic Peninsula (Washington State, USA): Descriptions and Functional Morphology

**DOI:** 10.1371/journal.pone.0025672

**Published:** 2011-10-31

**Authors:** Gareth J. Dyke, Xia Wang, Michael B. Habib

**Affiliations:** 1 School of Biology and Environmental Sciences, University College Dublin, Belfield, Dublin, Ireland; 2 Department of Sciences, Chatham University, Pittsburgh, Pennsylvania, United States of America; Raymond M. Alf Museum of Paleontology, United States of America

## Abstract

The plotopterids (Aves, Plotopteridae) were a group of extinct wing-propelled marine birds that are known from Paleogene-aged sediments (Eocene to Miocene), mostly around the Pacific Rim (especially Japan and the northwest coast of North America). While these birds exhibit a strikingly similar wing morphology to penguins (Spheniscidae), they also share derived characters with pelecaniform birds that are absent in penguins and exhibit apparently superficial similarities with auks (Alcidae: Charadriiformes). Despite quite an abundant fossil record, these birds have been little studied, and in particular their functional morphology remains little understood. Here we present osteological overviews of specimens from the northwest coast of Washington state (USA). We give an amended diagnosis for the well-represented North American genus, *Tonsala* Olson, 1980, describe a new large species, and examine the functional morphology of plotopterids showing that the ratio of humeral strength to femoral strength is quite low in one well-represented species *Tonsala buchanani* sp.nov., relative to both extant penguins and alcids. While the femoral strength of *Tonsala buchanani* is ‘penguin-grade’, its humeral strength is more ‘alcid-grade’. These results have implications for understanding the mode-of-locomotion of these extinct marine birds. Although not related to Spheniscidae, our descriptions and functional results suggest that *Tonsala buchanani* sustained similar loads in walking, but slightly lower humeral loads during swimming, than a modern penguin. This suggests a swimming mode that is more similar to living alcids, than to the highly-specialised locomotor strategy of living and fossil penguins.

## Introduction

The plotopterids (Aves, Plotopteridae) were a group of extinct marine birds that are known from Paleogene sediments spanning the northern Pacific rim [1]–[10]. These birds are particularly interesting not only because they are thought to have been exclusively marine, but also because they were also flightless and wing-propelled; plotopterids had an abbreviate wing that superficially resembles the ‘flipper’ of living and fossil penguins (Spheniscidae). First described from the early Miocene (ca. 18 Ma) of southern California by Howard [1] on the basis of a single cranial portion of coracoid [4], [11] these marine birds are now known from an array of specimens ranging in age as far back as the Upper Eocene (ca. 35 Ma); thus, the stratigraphic range of this unusual avian lineage encompassed at least 20 million years. Although well-described from the Japanese Pacific coast [5], [6], [10], few of the known North American plotopterid specimens have yet been discussed in detail; to date, just preliminary descriptions [7], [8] and overviews [11, this paper] have been presented. It is also true that the fossil record of Paleogene birds from the Pacific Northwest (western Canada and northwestern USA) remains little studied, in spite of the numbers of specimens housed in regional museum collections.

Howard [1] first noted anatomical similarities between the plotopterids and both Pelecaniformes (including darters and cormorants) and penguins. Subsequent descriptions from the northern Pacific coast of the USA (Washington State) [4] and Japan [3], [5], [6], [10] have led to the hypothesis that these birds are most closely related to extant Pelecaniformes, within the suborder Sulae [4], [10], [11], [12]. One alternative hypothesis, based on a cladistic analysis of 68 morphological characters [9], is that plotopterids are most closely related to penguins; anatomical similarities with birds placed in the traditional grouping of pelecaniforms thus representing basal character states (in Mayr's 2005 [9] hypothesis), as the three lineages are suggested to comprise the same clade (Mayr, 2005: p 63). Recently, a more comprehensive morphological study [13], has confirmed the more traditional view for the relationships of these birds: Smith's [13] analysis of more than 460 osteological characters postulates a sister-group relationship for plotopterids with Anhingidae and Phalacrocoracidae, another branch within Sulidae (Smith, 2010: figure 2).

Finally, although the plotopterid forelimb (particularly the distal end of the humerus) is superficially similar to flightless alcids, including the extinct Great Auk (*Pinguinus impennis*) [14] and the Miocene Mancalline, or Lucas Auks [15], this third hypothesis for the relationships of these birds has never been discussed.

In this paper, we present anatomical descriptions of North American plotopterid remains based on five incomplete (but associated) specimens collected from the mid-1980s onwards from the northern coast of Washington State (the Olympic Peninsula). These fossil birds come from sediments that border the Strait of Juan de Fuca (the Coast Range terrane of the Cascadian accretionary wedge) – the Eocene-Oligocene Makah Formation and the overlying Oligocene Pysht Formation [7] – and thus are among the oldest known records of plotopterids. We also describe a new large species within the genus *Tonsala* Olson, 1980. Given the unusual morphologies seen in these birds, we also address the relationship between their limb proportions and likely mode of locomotion by comparing them to several lineages of living wing-propelled diving birds.

### Institutional Abbreviations

UWBM, Thomas Burke Memorial Washington State Museum (Burke Museum), University of Washington, Seattle, USA; RBCM, Royal British Columbia Museum, Victoria, BC Canada; USNM, United States National Museum (Smithsonian Institution), Washington DC, USA.

## Results

### Systematic Paleontology

Aves Linnaeus, 1758 [16].

Neornithes Gadow, 1893 [17].

Plotopteridae Howard, 1969 [1].

#### Included generic-level taxa

*Plotopterum* Howard, 1969; *Tonsala* Olson, 1980; *Phocavis* Goedert, 1988; *Copepteryx* Olson and Hasegawa, 1996; *Hokkaidornis* Sakurai et al. 2008.

### *Tonsala* Olson, 1980

#### Diagnosis

The generic diagnosis provided for this taxon by Olson [4, p. 52] refers only to the cranial end of the coracoid, the only part of the skeleton available to Howard [1] for description of the Miocene plotopterid *Plotopterum*. Additional features unique to this taxon include the presence of marked pits (for the attachment of feather tracts) on the midshaft dorsal surface of the ulna (only known in the type of *Tonsala hildegardae*, Olson, 1980) and a markedly hooked and pointed processus procoracoideus.

### *Tonsala hildegardae* Olson, 1980

#### Holotype specimen

A partial skeleton (USNM 256518) from the lower portion of the Pysht Formation [4], close to Murdock Creek on the Olympic Peninsula of Washington state [18]–[21]. Measurements are in Table 1 ([4]).

**Table 1:** Measurements of selected Olympic Peninsula plotopterid bones (in mm) (partly based on [4] and [8]).

	USNM256518	UWBM86869	UWBM86870	UWBM86871	UWBM86873	UWBM86874	UWBM86875
Proximal width of humerus	27.9	—	—	30.6	—	—	—
Proximal depth of humerus	19	—	—	—	—	—	—
Distal width of humerus	22.7	35.6	—	27.6	13.8	—	—
Distal depth of humerus	13.3	—	—	—	—	—	—
Shaft width distal to palmar crest of humerus	16.8	25.6	—	21.2	—	—	—
Shaft depth distal to palmar crest of humerus	7.9	—	—	—	—	—	—
Total length of humerus	—	—	—	143.8	—	—	—
Distance from head to distal extent of glenoid facet of coracoid	41.8	46.4	—	42.2	—	—	—
Length of glenoid facet of coracoid	14.6	27.2	—	28.6	—	—	—
Breadth below head across triosseal canal of coracoid	12.7	13.8	—	18.6	—	—	—
Length of pterygoid	—	43.2	—	—	—	—	—
Length of mandible	—	87.2	—	—	—	74.2	—
Depth posterior of symphysis						6.6	
Medial width of mandible	—	13.6	—	—	—	—	—
Total preserved length of scapula	141.1	—	—	—	—	—	—
Width at narrowest point of scapula	10.7	—	—	—	—	—	—
Length of ulna	72.5	—	—	—	—	—	—
Proximal depth of ulna	18.7	—	—	—	—	—	—
Droximal width of ulna	12.5	—	—	—	—	—	—
Distance from distal end of metacarpal I to distal end of metacarpal II	24.8	36.8	—	—	—	—	—
Distal depth of carpometacarpus	14.4	—	—	—	—	—	—
Distal width of carpometacarpus	6	16.8	—	—	—	—	—
Length of intermetacarpal space	25.9	32.8	—	—	—	—	—
Greatest diameter of proximal articulation of radius	8.6	—	—	—	—	—	—
Length of sternal rib	—	—	—	—	57.9	—	54.4
Length of synsacrum	—	—	—	—	154	—	—
Width at acetabulum					38.4		
Total length of femur	—	—	135.1	—	106.5	—	134.2
Proximal width of femur	—	—	31.2	—	24.2	—	—
Distal width of femur	—	—	28.9	—	23.6	—	25.2
Shaft width of femur	—	—	14.4	—	11	—	—
Proximal width of tibiotarsus	—	—	—	—	18.7	—	15.6
Proximal width of tarsometatarsus	—	—	28.8	—	—	—	—
Length of metatarsal II	—	—	58.8	—	—	—	—
Length of metatarsal III	—	—	62.2	—	—	—	—
Width of trochlea for metatarsal II	—	—	10	—	—	—	—
Width of trochlea for metatarsal III	—	—	11	—	—	—	—

#### Referred specimens

**Figure 1:**
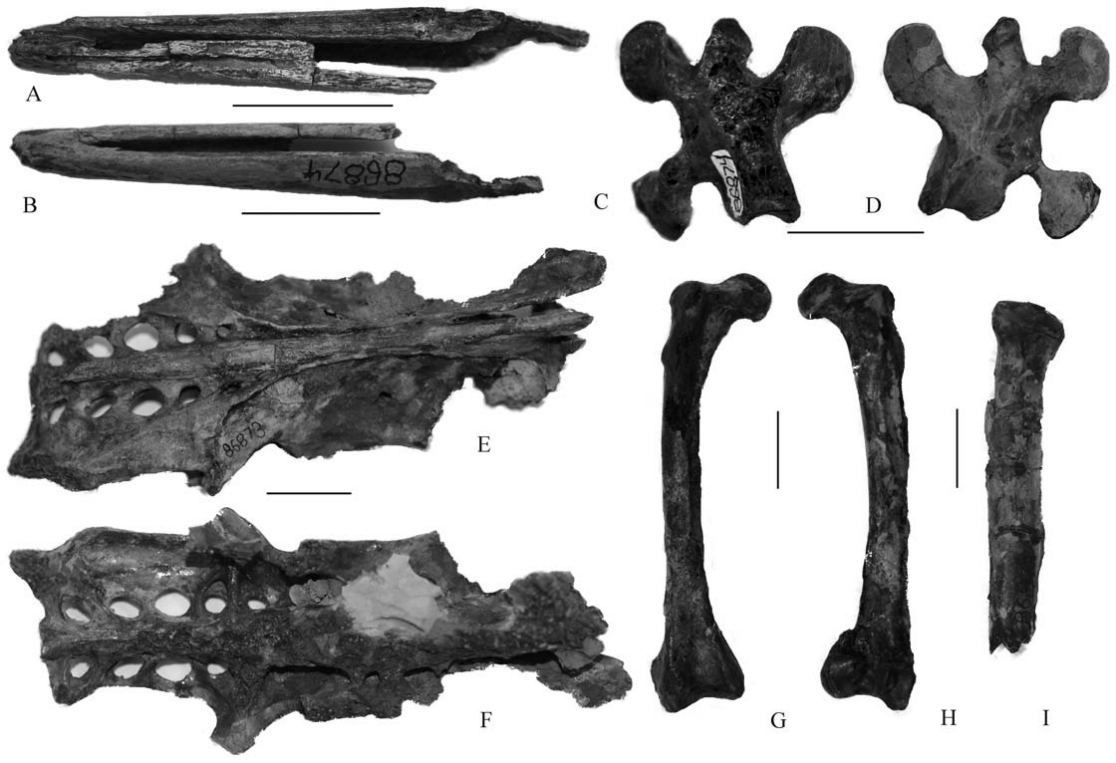
Fossil material referred to *Tonsala hildegardae*. A–D, dentary in dorsal (A) and ventral (B) views, UWBM 86874. C–D, cervical vertebrate in dorsal (C) and ventral (D) view, UWBM 86874. E–F, pelvis in dorsal (E) and ventral (F) view, UWBM 86873. G–H, right femur in cranial (G) and caudal (H) view, UWBM 86873. I, right tibiotarsus in caudal view, UWBM 86873. Scale bar is 2 cm.

UWBM 86873 and UWBM 86874 (Figure 1), briefly described and referred by Goedert and Cornish [8]. Both these specimens were collected by J. and G. Goedert from Murdock Creek (Pysht Formation) and include a selection of fore and hindlimb bones [7] as well as portions of the pelvis and the anterior region of an isolated dentary (UWBM 86874). Our referral to *Tonsala* and to *T. hildegardae* is based on comparisons of the overlapping proximal humerus with the holotype, USNM 256518 ([8], p. 69). While USNM 256518 comprises mostly forelimb elements, UWBM 86873 and UWBM 86874 are largely represented by bones from the pelvis and legs.

#### Diagnosis

The diagnosis provided for this taxon by Olson ([4], p. 52) is the same for the genus. Additional features, besides characters mentioned for the genus, are as follows: fossa m. brachialis of humerus deep and rounded in caudal view; margin between crista brachialis and humeral shaft greater than 90 degrees; sulcus ligamentous transversus does not reach the impressio coracobrachialis; processus extensorius of carpometacarpus rounded, blunt and upturned; fossa infratrochlearis shallow above processus pisiformis; cranial margin between trochlea carpalis and processus pisiformis very wide.

### *Tonsala buchanani* sp. nov

#### Holotype specimen

**Figure 2:**
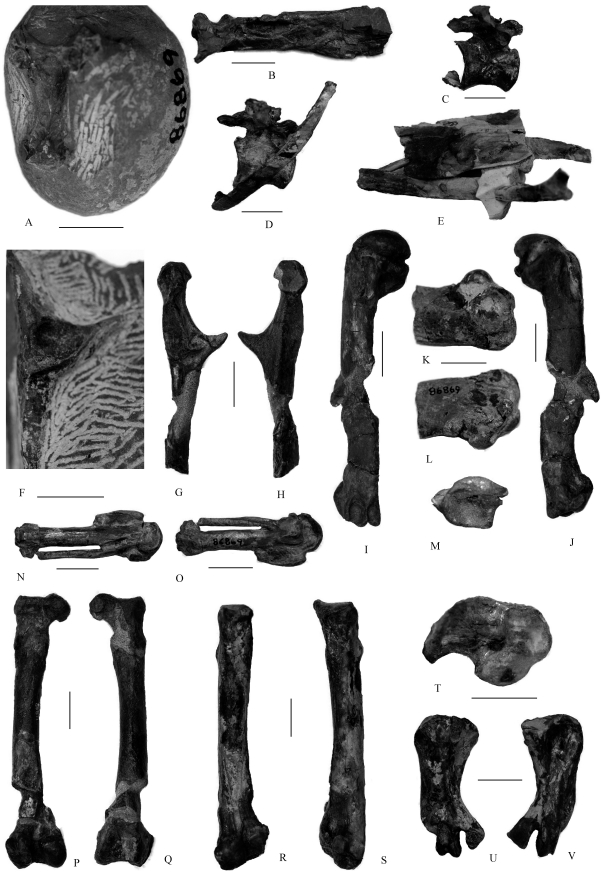
Fossil material referred to *Tonsala buchanani*. A, Pterygoid in ventral view, UWBM 86869. B, left mandible in ventral view, UWBM 86869. C–E, thoracic vertebrae with ribs and pars hepatica of sternum side view, UWBM 86875. F, left coracoid in dorsal view, UWBM 86869. G–H, left coracoid in dorsal (G) and ventral views (H), UWBM 86871. I–J, left humerus in cranial (I) and caudal (J) views, UWBM 86871. K–L, distal left humerus in cranial (K) and caudal view (L), UWBM 86869. M, radius in proximal view, UWBM 86871. N–O, right carpometacarpus in ventral (N) and dorsal (O) view, UWBM 86869. P–Q, right femur in cranial (P) and caudal (Q) view, UWBM 86871. R–S, right (R) and left (S) femur in cranial view, UWBM 86875. T, tibiotarsus in proximal view, UWBM 86871. U–V, left tarsometatarsus in ventral (U) and dorsal (V) view, UWBM 86870. Scale bar is 2 cm.

UWBM 86875 (Figure 2), a partial skeleton comprising distal ends of both femora (the caput of the left is associated but broken), proximal articular surface of left tibiotarsus, three thoracic vertebrae (one with associated ribs) with intact transverse processes, caudal end of sternum, right fibula, proximal end of right tarsometatarsus and a number of additional broken rib fragments with their costal processes intact.

#### Etymology

Named for William “Bill” Buchanan (deceased), formerly of Clallam Bay, Washington, for the many specimens he collected, helped collect, and donated to West Coast natural history museums.

#### Referred specimens (Figure 2)

Based on size and comparisons of overlapping skeletal elements: UWBM 86870, left tarsometatarsus lacking trochlea for metatarsal IV; UWBM 86871, partial skeleton comprising complete right femur (glued together in the UWBM collection back-to-front), complete right humerus, distal end of left humerus, proximal articulation of left radius, cranial end of left coracoid (broken at the level of the sternocoracoid impression) and some broken pieces of ribs; UWBM 86869, partial skeleton comprising the posterior portion of a left mandible (including the retroarticular process), distal end of right humerus, right carpometacarpus (lacking distal end of os metacarpale minus), cranial portion of left coracoid, part of one pterygoid, and one incomplete cervical vertebra.

#### Locality

The holotype (UWBM 86875) was collected from the same area as the holotype of *T*. *hildegardae* [4]: Pysht Formation outcrop to the west of Murdock Creek, on the Olympic Peninsula of Washington State. UWBM 86870 (Figure 1) was collected from the Lower Oligocene portion of the Makah Formation, southeast of Jansen Creek while UWBM 86871 was collected southeast of Bullman Creek (Makah Formation). UWBM 86869 also comes from the mid-portion of the Makah Formation exposed at Whiskey Creek on the Olympic Peninsula. All three specimens were collected by J. Goedert.

#### Diagnosis

The characters listed here differentiate *Tonsala buchanani* from its counterpart, *T. hildegardae* Olson, 1980. Humerus: Shallow fossa m. brachialis in caudal view (cf. USNM 256518, where this fossa is deeper and more rounded); margin between crista brachialis and humeral shaft almost 90 degrees (this angle is markedly greater than 90 degrees in USNM 256518; Olson, 1980: fig. 2); sulcus ligamentous transversus extends all the way across the cranial surface of the proximal humerus (this sulcus does not reach the impressio coracobrachialis in USNM 256518). Coracoids much larger in size (Table 1). Carpometacarpus: processus extensorius rounded and blunt (not upturned as in *T. hildegardae*); os metacarpale alulare extends distally down the length of the element as a flat shelf; fossa infratrochlearis of carpometacarpus deep above processus pisiformis (this region not as excavated in *T. hildegardae*); cranial margin between trochlea carpalis and processus pisiformis narrow (wider in *T. hildegardae*).

#### Remarks

Three specimens, UWBM 86869, UWBM 86870 and UWBM 86871, were referred to Plotopteridae by Goedert and Cornish [8], who noted their distinctively larger size in comparison to *T. hildegardae*. As discussed by Goedert and Cornish [8], the corresponding elements of this larger species of *Tonsala* are about twice the size of those of *T. hildegardae* (Table 1); because these bones are all from mature individuals, at least in comparison with extant birds, the possibility of distinct size-classes of a single species can be ruled out. Though some sexual size dimorphism exists in both Pelecaniformes and Spheniscidae, obvious differences in body size between sexes is only seen in Phalacrocoracidae [22]; thus, although possible, the likelihood of sexual dimorphism being a factor in interpretation of these specimens is slim. Other than size (Diagnosis), however, bones of *T. buchanani* are anatomically very similar to those known for *T. hildegardae*; salient anatomical features of both species are thus presented in the descriptive sections below. Descriptions of some forelimb bones (ulna, radius) as well as the scapula are partly based on the *T. hildegardae* holotype (USNM 256518) [4].

### Skull and mandible

Very few skull bones are represented in the UWBM collection of plotopterids from the Olympic Peninsula [8]. The left pterygoid that forms part of UWBM 86869 -is short and expanded anteriorly with a depressed medial portion, a feature also seen in extant penguins [23]. At its posterior end, there is a shallow concave cotyle for articulation with the quadrate. The anterior end bears a semicircular articular facet for the parasphenoid rostrum and a small projection for articulation with the right pterygoid (Figure 2A). The two dentary portions (UWBM 86869 and UWBM 86874) are different sizes and come from the caudal (UWBM 86869) and rostral (UWBM 86874) regions of the skull, respectively. The ventral surface of the mandible is separated, unossified and ungrooved (UWBM 86874), while the dorsal margin is straight, with a shallow groove of similar depth for much of its length that tapers anteriorly (Figures 1A, B and 2B). In lateral view, the dentary is a wide, robust and flat plate with a poorly-developed angulus medialis (Figure 1A, B): A short symphysis is preserved here, however, whether there is any extension is unclear due to the bone's incompleteness. The processus mandibularis medialis (UWBM 86874) is well-developed, broad and somewhat retroverted caudally, similar to the condition seen in penguins [23]; the cotyla lateralis is broad and open as an angled groove. An elongate and broad rostral mandibular fossa occurs on the internal surface but does not perforate the mandible. A caudal mandibular fossa occurs distally on the medial face of the mandible.

### Vertebrae and ribs

The third cervical vertebra is preserved in UWBM 86874 (Figure 1C, D); no bony bridge occurs between the processus transversus and the processus articularis caudalis. Thoracic vertebrae are preserved in the holotype (UWBM 86875). The preserved thoracic vertebrae are opisthocoelous; there are no marked lateral excavations on these elements or any sign of pneumatization, as is common for aquatic birds. ([8]). These vertebrae are large, with well-rounded centra and well-developed transverse processes. Although broken, their neural spines were broad and elongate. A number of isolated ribs (and associated fragments) are preserved as part of the Olympic Peninsula plotopterid collection in both UWBM 86874 and UWBM 86875; many of these elements have large and broad uncinate processes fused to them.

### Coracoid

Coracoids are preserved as part of the holotype of *T. hildegardae* (USNM 256518) and are also seen in UWBM 86869 and UWBM 86871 (Figure 2F–H). This bone is unusual in plotopterids amongst Neornithes in the angularity of the processus procoracoideus – according to Olson [4], formed as ‘a large anteriorly curved spine’, which is more similar to Sulidae and much larger than in the Anhingidae or Phalacrocoracidae – and in the absence of a caudally orientated (‘hooked’) processus acrocoracoideus. Seen in three-dimensions in UWBM 86871, this element is large, robust (very solid without any pneumatization; seen in broken portions of UWBM 86869), and has a deep, well-developed and ‘cup-like’ cotyla scapularis. The caput (processus acrocoracoideus) is large and rounded cranially (not turned caudally) and is offset laterally with respect to the processus procoracoideus; the sulcus m. supracoracoideus is broad, flat and well-rounded. In medial view, the facies articularis clavicularis is large, well-rounded and bulbous (this facies does not overlap the sulcus supracoracoideus). There is no foramen nervi supracoracoidei on the dorsal coracoid surface. The facies articularis humeralis is turned obliquely onto the dorsal face of this element.

While the caudal end of the coracoid is unknown for North American plotopterids [1], [4], preservation of UWBM 86871 shows that the caudal portion of this element was turned obliquely; this bone was long, thin and narrow.

### Scapula

The scapula of *Tonsala* is known only from the holotype of *T. hildegardae*, USNM 256518 [4]. This element is much like that of *H. abashiriensis* in that it is very thin and has a markedly expanded, almost ‘rudder-like’ facies lateralis, a character ‘unlike that of any other birds except penguins’ [4]. The margo dorsalis and extremitas caudalis are both weathered away in USNM 256518, while the caudal margin of this element is not curved or tapered. The acromion is very large and markedly pointed, extending rostrally well beyond the level of the the tuberculum coracoideum. The facies articularis humeralis is flat and extended laterally, resembling the condition seen in Pelecaniformes, and is distinct from the morphology in Sphenisciformes and Charadriiformes [4].

### Humerus

An almost complete and well-preserved humerus is known for UWBM 86871 (Figure 2I–L). This is a large element (Table 1) with a robust, very flat and straight shaft; the proximal and distal ends are not markedly offset from one another. In contrast, the shaft of this bone is more sinusoidal in *T.hildegardae* and *H. abashiriensis* [10]. Portions of proximal humeri comprise part of the holotype of *T. hildegardae* (USNM 256518) as well as UWBM 86873. The proximal end of this element is very heavy [4], with a well-rounded, almost spherical caput that is ventrally directed, most similar to penguins. This was noted by Olson [4], who regarded this character as a feature shared only with penguins among known birds. The margin of the head is not projected ventrally (see Mayr, 2005; character 36), as in many neornithine birds [24]. The incisura capitis is deep and wide, extending all the way across the proximal caudal surface of the humerus above a marked, rounded (but not pnuematized) crus dorsale fossa. This fossa is turned obliquely into the margin of the shaft, so that it is only slightly visible in caudal view (Figure 2J). Above this fossa, the tuberculum ventrale is well-developed as a knob, separated from the lateral margin of the dorsal fossa by a distinct notch.

On the cranial surface, the sulcus ligamentous transversus is also deep, wide and well-marked, extending across the entire body of the proximal humerus (Figure 2I). Unusually, the crista bicipitalis is ‘wedge-shaped’ and flat, raised cranially away from this marked sulcus, as in the humerus of the Miocene alcid *Mancalla* [15]. The crista deltopectoralis is short, narrow and angular, extending only a very short distance down the cranial margin of the humerus; the margin between this crest and the shaft is imperceptible. The impressio coracobrachialis in UWBM 86871 is also marked; forming a semi-circular outline on the cranial surface (Figure 2I).

The humeral shaft of *Tonsala* is flat, compressed craniocaudally as in penguins and flightless auks (Alcidae). On its distal end, this flattening is extreme such that the entire shaft is upturned, creating a distinct ‘flick’ to the articulating surface in caudal view (Figure 2I–L) [9]. However, this flattening and modification of the distal end occurs to a lesser degree than is seen in modern penguins and as such more closely resembles the condition in the flightless mancalline auks (Alcidae), for example *Mancalla* Lucas [6], [15], [25]. Distally, the body of the humerus is projected ventrally such that the sulci scapulotricipitalis and sulci humerotricipitalis form two deep, marked caudal furrows bordered by pronounced ridges (Figure 2I–L), also similar to the condition seen in *Mancalla* [25] and penguins [23] and shallower than that of *Copepteryx* [6]. The processus flexorius is absent and the fossa olecrani is obliterated in *Tonsala*. In cranial view, the fossa m. brachialis is shallow in UWBM 86871, bordered by the remnants of the distal humeral condyles, again as in *Mancalla* and penguins. This depression is deeper and more marked in USNM 256518 which closely resembles the alcid *Pinguinus* in this regard. A faint tuberculum supracondylare ventrale also occurs on the distal humerus of UWBM 86871 (Figure 2I, K).

A well-preserved humeral distal end is also preserved in UWBM 86869. The distal ends of the humeri of UWBM 86869 are larger than in UWBM 86871 and are similar in size to humeri of *Copepteryx hexeris*. Although the humerus shows typical characters of *Tonsala*, this bone differs from UWBM 86871 and *Copepteryx hexeris* in details of the shape and development of the condyles (see [8]). UWBM 86869, from strata near Whiskey Creek, is also older - probably late Eocene - than either the Jansen Creek Member fossils (Early Oligocene) or those from near Murdock Creek (Early Oligocene) [8]. While UWBM 86869 may belong to a new species of *Tonsala*, more complete specimens will be needed for its further identification. Here, we tentatively refer it to *Tonsala buchanani*.

### Ulna and radius

The ulna and parts of the radius of *Tonsala* are only preserved in the holotype of *T. hildegardae* USNM 256518 ([4]: fig. 3); these bones are greatly expanded and flattened, but less so than in modern penguins [26]–[28]. These elements of *Tonsala* strongly resemble the corresponding elements of *Waimanu*, a Late Paleocene penguin figured by Fordyce and Jones [26], [27] and Slack et al. [28].

The ulna in plotopterids is quite unique – a straight foreshortened element (probably shorter than the corresponding humerus, but complete elements are not preserved in the same specimen) that bears marked pits for the attachment of the secondary feathers [4] on the middle of its dorsal surface. A similar row of pits, less marked, is found in some modern penguins [9]. The proximal end of the ulna is not compressed or inflected; this is a bulbous and rounded surface. The shaft of the ulna (USNM 256518) is broad: this bone tapers from a broad and wide proximal end with a rounded olecranon to a narrow and rounded distal surface that does not bear a marked depressio radialis. According to Olson [4], the internal cotyla is quite large and deep, but the external cotyla is so modified as to be convex in proximal view.

Corresponding with this ulna, the remains of the radius of USNM 256518 show that this was a flat element very similar to that of *C. hexeris* [6], *H. abashiriensis* and the flightless Miocene alcid *Mancalla*. The proximal articulation of the radius in UWBM 86871 (*T. buchanani*) shows that the cotyla humeralis was depressed in these birds and confirm that the shaft was broad and flat (Figure 2M). In general, the radius and ulna in *Tonsala* are neither as flattened as penguins nor as shortened as in *Mancalla*.

### Carpometacarpus

Incomplete portions of proximal carpometacarpi are preserved as part of the holotype of *T. hildegardae* and in UWBM 86869 (Figure 2N,O). A weathered portion of a distal articulation is seen in UWBM 86873 (referred to *T. hildegardae*; [8]). The carpometacarpus of UWBM 86869 is similar to that of *C. hexeri* but is shorter [8]. In both species of *Tonsala* this element is flattened; the trochlea carpalis is pronounced and well-rounded, a fossa infratrochlearis occurs (variably marked) and the processus pisiformis is pronounced, whereas in *Mancalla*, the processus pisiformis is obsolete [25].

The os metacarpale majus is broad, robust and straight, while the os metacarpale minus is much thinner and curved (Figure 2N,O); the spatium intermetacarpale is narrow, long and thin. The proximal end of the os metacarpale minus is narrow and is not deflected ventrally. A pronounced groove occurs between the two facets of the facies articularis digitis major on the distal articulation (UWBM 86873 and UWBM 86869).

### Pelvis and sternum

Well-preserved examples of plotopterid pelves are part of UWBM 86873 and UWBM 86874, both referred to *T. hildegardae* [8]. The pelvis is long and narrow in this taxon, at least compared to the corresponding region in Japanese plotopterids (*Copepteryx*) [6]. In dorsal view, the anterior iliac shields of *Tonsala* are not expanded as in most Sulidae, Anhingidae and Phalacrocoracidae, while the pre- and postacetabular regions are approximately equal in length, as in *Copepteryx*, unlike the elongate postacetabular pelvis that is seen in Sulidae and Phalacrocoracidae (Figure 1E). Compared with *T. hildegardae*, the median ridge of the pelvis of *H. abashiriensis* is concave anteriorly (rather than convex), and the anterior iliac crest is more widely divergent anteriorly. In these regards, this bird was considered most similar to *Anhinga* spp. [10].

UWBM 86873 and UWBM 86874 come from almost identically sized birds (Table 1); however, pubes are not preserved in either specimen. Because both are also broken cranially, the number of synsacral vertebrae cannot be determined: nevertheless at least ten vertebrae comprise this region in UWBM 86873 (Figure 1E, F). The cristae iliacae dorsales are open cranially.

Six pairs of large foramina (*foramina intertransversariae*) are preserved on the dorsal surface of the synsacrum in UWBM 86873 and are also seen in *H. abashiriensis*; these perforate the postacetabular area as is in many charadriiforms [29].

Also as in alcids, the degree of ossification between transverse processes of adjacent vertebrae is reduced and the synsacrum is heavily perforated by large fenestrae. In lateral view, the foramen ilioischiadicum is open, long and narrow in *Tonsala* (UWBM 86873) and the foramen acetabuli is not enclosed by the corpus ischii. The crista iliaca dorsalis does not overlap the lateral margin of the ilium (no marked shelf is formed) and is not raised dorsally – this is a flat shelf rather than a raised ridge (Figure 2E). In ventral view, a marked boss (“synsacral strut”) formed by the vertebral costal processes occurs between the mid-section sacrals and the lateral margins of the ilium (Figure 1F), a character present in charadriiformes generally, with the exception of alcids [29]–[33].

Only one side of the sternal carina (pars hepatica) is preserved as part of UWBM 86875: this is a wide and flat plate (Figure 2E). Olson regarded the character ‘sternum with large, pointed carina projecting far anterior to coracoidal sulci’ as evidence for monophyly of Plotopteridae and Pelecaniformes to the exclusion of Spheniscidae [4], while according to Mayr, this character doesn't distinguish plotopterids from penguins in which the apex carinae points markedly cranially [9].

### Femur

The femur is the best represented and most robust element preserved in the Olympic Peninsula plotopterid collections, including UWBM 86871(left), UWBM 86875 (left), and UWBM 86873 (right) (Figure 1).

The femur of *Tonsala* is much smaller, less robust and proportionately more elongate than *H. abashiriensis* and more similar to *Plotopterum* sp. [5]. The shaft of *Tonsala* is curved in medial view (Figure 2P, Q), unlike *Copepteryx* and Anhingidae, in which the shaft is relatively straight. The femur of *Tonsala* also differs from the same element in *H. abashiriensis* and is similar to the femur of *C. titan* [6] in that it has a less bulbous head, a thinner and longer neck (especially in UWBM 86873), a less well-developed trochanteric ridge, a narrower and shallower intercondylar fossa and a narrower external condyle. Some of these characters (i.e., femur with proximal and distal ends proportionately broader, neck elongate) more closely resemble the conditions seen in birds placed together in the traditional grouping of pelecaniforms [4], [15], not penguins. However, the femur of the plotopterid *Copepteryx* very closely resembles that seen in some early Tertiary penguins [see 6].

### Tibiotarsus and fibula

Proximal parts of tibiotarsi (including pieces of shaft) are preserved as part of UWBM 86873 (*T. hildegardae*) and UWBM 86875; the shaft of this element is straight as well as broad, flat and compressed laterally [8]. The fossa flexoria, underneath the interarticular area, is well-developed in these birds, but the proximal surface is small and unexpanded. Both cristae are weakly developed and do not project proximally. The well-preserved proximal articulation of *T. buchanani* (UWBM 86875) shows that the facies articularis medialis is flat and that the area interarticularis is raised proximally as a bump (Figure 2T). In posterior view, the proximal articular surface of the head slopes less steeply mediolaterally than in does in *H. abashiriensis*. The crista patellaris is pronounced, although only slightly hooked. The incisura tibialis is wide. One fibula is also preserved as part of UWBM 86873; its proximal articulation with the tibiotarsus would have been flat, not overlapping the lateral margin of the shaft [8]. The proximal surface of the head has an anteroposterior ridge separating two long and shallow cotylae in *T. hildegardae*, whereas in *H. abashiriensis* the articular surface makes a smooth transition from the internal surface to the external edge [10]. Although broken, this element would not have extended more than one-half the total length of the tibiotarsus; its distal end is markedly tapered, almost to a point.

### Tarsometatarsus

Among the Olympic Peninsula tarsometatarsi, UWBM 86870 is the best preserved (Figure 2U,V). This bone is complete in three dimensions, lacking only the trochlea for metarsal IV. This element, like all the limb bones of these aquatic birds, is robust and compact; the tarsometatarsus is greatly abbreviated, measuring only about one quarter of the length of the tibiotarsus, similar to penguins, and much more abbreviated than the tarsometatarsus of all Suloidea. Comparative illustrations of this element were also provided by Goedert and Cornish ([8]: fig. 3); in plotopterids, this bone is stout and robust, completely fused and compressed somewhat proximodistally, most like the condition seen darters, bearing little resemblance to the morphology of modern penguins, in which the metatarsals are incompletely fused. The tarsometatarsus of penguins from the early Tertiary is similar to that of Plotopteridae [26]–[28]. Goedert and Cornish [8] noted that in the larger plotopterids (for which this element is known) the tarsometatarsus tends to be a broader and more splayed element; this is likely directly related to an increase in body size [8].

On the proximal surface of the tarsometatarsus (UWBM 86870) a single robust crista medialis hypotarsi occurs, extending somewhat ventrally (Figure 2U,V). A crista lateralis hypotarsi is also present, but is small and weakly projected; consequently, a broad sulcus that extends proximally to the hypotarsal surface is present between these crests (but not enclosed as a bony canal). On the proximal end of the tarsometatarsus two foramina occur (Figure 2V). While the medial formen is larger (elongate and ovate in shape), its smaller lateral counterpart perforates the shaft completely and emerges onto the ventral surface (Figure 2U). Because the lateral margin of UWBM 86870 is upturned proximally, the margin of this foramen extends higher up the dorsal surface in this element. Indeed, both cotylae are turned somewhat onto the dorsal surface of the shaft in this specimen; the cotyla medialis extends further distally and is wider. The eminentia intercotylaris is smoothly rounded and not markedly pronounced (Figure 2U,V).

In dorsal view (Figure 2V), trochlea metatarsi II is deflected somewhat plantarly but is extended as far distally as the estimated extent of the fourth (i.e., trochlea metatarsi IV is elevated well above the others while trochlea metatarsi II is elongate and at same level as is the middle trochlea; this is similar to the condition in Pelecaniformes and Tertiary penguins [4], [9]). A foramen vasculare distale, reported in *Phocavis maritimus*, also occurs on UWBM 86870, completely enclosed medially with respect to trochlea metatarsi III; this was described as ‘foramen vasculare distale distally open or completely absent’ by Mayr [9]. While trochlea metatarsi II is smooth and flat on its distal surface, trochlea metatarsi III is markedly grooved; the medial margin of this trochlea extends further distally than does its lateral margin (Figure 2U,V), similar to the condition seen in some galliforms [34]. The incisura intertrochlearis medialis is broad and deep. The medial margin of trochlea metatarsi III is perforated by a small proximal fossa.

### Limb strength in plotopterids

Analysis of the limb elements referrable to *Tonsala buchanani* indicate an interesting mixture of structural characters relative to living wing-propelled diving birds. The structural strength of the forelimb elements of wing propelled divers, especially penguins, is typically quite high [35], [36].

Estimates of the structural strength (i.e. section moduli) for the humeri and femora of several living species of wing-propelled divers, along with two species of *Tonsala*, are given in Table S1. Section moduli are here estimated as if the bones were solid, because cortical breadth data were not available for the plotopterid specimens. Section modulus varies by the cube of the distance from the neutral axis of a section. This means that the inner layers of bone add very little to strength, and that very thick-walled bones can be approximated as solid sections with limited loss of precision. The penguins and alcids in the dataset were previously subjected to CT scans to obtain cortical area data. The solid section estimate errors for these specimens vary from 0.1% (*Aptenodytes forsteri*) to 5% (*Cerorhinca monocerata*). Plotopterids possessed thick-walled bones [3], [9], and therefore solid section estimates also closely approximate their structural strength.

Although arguments exist concerning the safety factors for avian bones [37], [38], these do not affect our comparative analysis below, so a consistent safety factor is assumed for all specimens. The ratio of humeral Z_y_ to humeral Z_x_ (bending about the x and y axes) indicates that the humerus of *Tonsala buchanani* is less flattened than in penguins, but relatively more flattened than in alcids (Table S1). The ratio of humeral strength to femoral strength (which is mass-independent) is quite low in *Tonsala buchanani*, relative to both penguins and alcids. In penguins, a high humeral to femoral strength ratio is largely the product of rigid forelimb elements. In alcids, a similar ratio exists but it is more dependent upon their weak femora [39].

In *Tonsala buchanani*, it appears that femoral strength is penguin-grade (1.15 standard deviations from the mean for penguins, 2.91 standard deviations from the mean for alcids), but humeral strength is more alcid-grade (2.67 standard deviations from the mean for penguins, 0.76 standard deviations from mean for alcids). Body mass for *T. buchanani* was estimated by using a regression of humeral length against body mass for living aquaflyers. A similar result (0.25 kg lighter) is obtained using femoral length. Because the estimates of structural strength are length-corrected, only element breadth is non-independent in this comparison (meaning that *T. buchanani* has relatively wide femora and a narrow humerus, compared to penguins). The overall mass-specific strength relationship holds, however, even if *T. buchanani* is substantially lighter or heavier than estimated. As a result, the general mass-specific strength relationship recovered is robust even if the ratio of humeral or femoral length to body mass was differed slightly in plotopterids as compared to other aquaflying birds. Given that its limb elements are significantly longer than those for the largest living penguins (*Aptenodytes*), it seems reasonable that the body mass for *Tonsala buchanani* should be somewhat greater than for any living penguins. Therefore, the somewhat tentative calculation from humeral length (30.88 kg) is considered a plausible rough estimate.

## Discussion

### Locomotion

We propose that the ‘penguin-like’ femoral strength (length-corrected polar femoral section modulus) of plotopterids is indicative of extensive terrestrial locomotion, as in living penguins. This is an expected result because plotopterids are thought to have been largely marine, flightness birds (as penguins are today). The ‘alcid-type’ humeral strength (length-corrected polar humeral section modulus) in bending recovered by our analysis is, however, more difficult to interpret, but likely indicates that the stroke pattern of *Tonsala buchanani*, during swimming, less mirrored than that of living penguins, and incorporated a greater amount of surge acceleration between half-strokes, as is seen in living alcids. While alcids do swim with an upstroke that results in some useful lift generation, the relative contribution to thrust is substantially less than that achieved by penguins [40]. Penguins differ in this regard; they generate substantial thrust on both upstroke and downstroke, essentially eliminating a true ‘recovery’ phase [40], [41]. As a result, penguins have effective power stroke frequencies nearly double that of aerial flyers using the same overall wing-beat frequency. Penguins possess considerably stronger forelimb elements than alcids, even when corrected for body size and scaling effects [36].

Living alcids do not possess humeri with greater strength in bending than other flying birds, while penguins possess greatly reinforced humeri compared to all other living birds. Both alcids and penguins possess thick-walled bones, which provide increased structural strength and ballast. However, the humeri of penguins are also exceptionally short and deep (wide in the craniocaudal plane, especially), which is a purely structural characteristic [36]. The precise nature of the structural loading regime in penguins during swimming has not been measured *in vivo*, but comparative work suggests that penguins may develop greater maximum stress within their forelimb skeletal spar during their particularly powerful stroke reversals [36], which are a by-product of the use of the upstroke as a major contributor to thrust [40], [41]. This mirrored stroke reduces surge acceleration inefficiency [39], but likely comes at the cost of greater bending loads and a concomitant need for greater structural reinforcement.

Based on our structural analysis, we suggest that *Tonsala buchanani* sustained similar loads in walking to living penguins, but sustained slightly lower humeral loads during swimming than penguins do. If the substantial reinforcement of the penguin forelimb skeleton is indeed related to a mirrored swimming stroke cycle (specifically, to the associated high-force stroke reversal phases), then *T. buchanani* likely swam in a manner somewhat more similar to living alcids than to living penguins, with the downstroke producing the majority of the propulsion. If this is true, then it might also imply that the relative mass of the supracoracoideus in *Tonsala* was similar to the supracoracoideus muscle fraction in alcids, which could have implications for sternal size and structure in plotopterids. As in alcids and penguins, the thickened bone walls of plotopterids [3], [9] may have been an adaptation for ballast.

Olson and Hasegawa ([3], p.688) described Plototeridae as “giant, flightless penguin-like birds” whose “hindlimb and pelvic morphology is most similar to that of recent darters, but the wing is paddle-like and remarkably convergent toward penguins and flightless auks.” According to Warheit [42], many Plototeridae were larger than penguins, possibly twice the height of the largest extant penguin. Three characters in Mayr [9] are not found in any other avian taxon except plotopterids and penguins: scapula forming a thin, sheetlike, greatly expanded blade; proximal end of humerus with a deep, rounded head and a ventrally directed caput humeri; os carpi ulnare flattened, with large caudal expansion. Plotopteridae and Spheniscidae further share highly derived tarsometatarsal morphology, whereas this bone is very different in all other wing-propelled diving bird including the Lucas auks [43]. On the other hand,the characters of the *T. buchanani* humerus show many similarities to Alcidae including *Mancalla* which was inferred to possess a penguin-like swimming flipper rather than an organ of potential flight that could be folded away in typical bird fashion when its owner came to rest [43].

Quantitative analyses of functional morphology shown that underwater modes of locomotion are reflected in skeletal features [44]. Considering the characters mentioned above and the limb strength, it is likely that these characters are related to a special mode of wing-propelled diving that penguins and plotopterids inherited from a common ancestor, rather than simply wing-propelled diving or it is also possible that *T. buchanani* may have swum in a manner intermediate between living alcids and penguins.

### Evolution of seabirds

Throughout the evolution of birds a number of lineages of ‘seabirds’ have occupied niches in the marine realm [15], [45].

The first known clade of exclusively aquatic birds were the foot-propelled Hesperornithiformes (e.g., *Hesperornis*, *Baptornis*, *Asiahesperonis*), known almost exclusively from marine and marginal marine sediments in the northern hemisphere up until the end of the Cretaceous (e.g., [46]–[47]). Following the Cretaceous-Paleogene (K-Pg) extinction, surviving lineages of marine birds within Neornithes included the late Paleocene to Pliocene-aged pseudodontorns and the Eocene-to-Miocene-aged plotopterids, both extinct lineages that filled niches presumably now occupied by taxa such as penguins and auks. Some workers have even suggested that marine birds, like plotopterids, may have been out competed by mammals in the mid-to-late Miocene [5], [45].

In any case, while penguins are known from a fossil record restricted to the southern hemisphere (e.g., [26]–[28]) that dates back to the Lower Eocene (ca. 55 Ma), auks (Alcidae) comprise a much younger radiation, dating from around the demise of the plotopterids in the Mio-Pliocene [45], [48]–[49]. While our current understanding of the fossil record would seem to indicate that plotopterids and pseudodontorns exploited marine niches in the northern Pacific until at least the mid-Miocene [43], no contemporary radiation of large numbers of Atlantic seabirds is known until the diversification of auks (Alcidae), albatrosses and mollymawks (Diomedeidae) in the Mio-Pliocene [45]. Factors controlling the evolution and suvivorship of seabirds remain poorly understood (and an area for future research), but it is likely that a combination of climatic change, oceanic temperature and the diversification of marine mammal lineages at this time all had a part to play in the extinction of the wing-propelled plotopterids [45].

## Materials and Methods

For geological information on sites, localities and maps, see Goedert and Cornish and Kiel and Goedert [8], [18]. Our use of anatomical terminology largely follows Baumel and Witmer [50] with some modifications to English, where appropriate.

For our bone strength analyses, structural strength in bending was estimated with a high degree of precision using measurements of the section modulus (Z), which measures the distribution of bone (or any material) about the neutral axis of bending in any given plane (the polar section modulus is the sum of any two orthogonal section moduli estimates through a given section). Bending and torsional loadings predominate in vertebrate limb bones [39], [51]–[54]. Bone strength can be defined as the inverse of maximum stress under loading, and represents the resistance to bending under any given load. Applying a beam model to vertebrate long bones, maximum stress in bending is given by 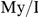 (where M is the bending moment, I is the second moment of area about the neutral axis, and y is the maximum distance from the neutral axis to the edge of the section) [55]. The section modulus, Z, in bending is defined as I/y. Taking M to be proportional to the product of body mass (B) and bone length (L) (femoral or humeral) [56]–[58], we are given the result that strength ∝ Z/(B*L). Modeling the midshaft as a true ellipse yields a simple formula for the calculation of polar section modulus (Z_p_):
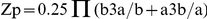
where ‘a’ and ‘b’ are the radii of the ellipse in any two perpendicular planes. For this study, ‘a’ and ‘b’ were taken as the dorsoventral and anteroposterior directions, respectively. The polar moment is the sum of two orthogonal moments. For our sample, Z_x_ is the bending strength in the dorsoventral direction, and Z_y_ is the bending strength in the craniocaudal direction. This formula is exact only for symmetric sections, but it is a strong approximation when the section closely approaches a true ellipse, which all of the measured avian elements do at their midshaft (the measured location for each bone). All values of comparative bending strength we report are based upon the polar modulus (Z_p_). The above formula, as written, gives the section modulus for a solid section. Section modulus has the dimensions of linear measurement to the third power (reported as mm^3^, in the case of the present avian sample). Dividing this value by the product of the moment estimate (body mass * element length) provides an estimate of relative structural strength in cantilever bending, which we use as a comparative structural measure to quantitatively assess mechanical differences between species.

## Supporting Information

Table S1 Structural strength (section moduli) for humeri and femora of several living species of wing-propelled divers, along with two species of *Tonsala*.
(DOC)

## Acknowledgments

We thank Jim Goedert for his kindness, support, and for collecting the fossil specimens discussed in this paper. Caroline Strömberg and Greg Wilson very kindly hosted GJD in Seattle; Ron Eng and Courtney Richards provided access to collections and much assistance at the UWBM. Gary Kaiser, Joan Kerik (RBCM) and David Starr also provided a good deal of advice and support while comparative osteological collections were examined at the Royal BC Museum, Victoria (Canada) with the assistance of Gary Kaiser and Michael McNall. Finally, we thank three reviewers and PLoS ONE editors Leon Claessens and Andrew Farke for their helpful comments on versions of this paper.
