# Supplementary material for: Fossil Plotopterid Seabirds from the Eo-Oligocene of the Olympic Peninsula (Washington State, USA): Descriptions and Functional Morphology
Source: PLoS One. 2011 Oct 31;6(10):e25672. doi: 10.1371/journal.pone.0025672 (PMC3204969; doi:10.1371/journal.pone.0025672)
Supplement: Table S1 — Structural strength (section moduli) for humeri and femora of several living species of wing-propelled divers, along with two species of Tonsala. (DOC) [file pone.0025672.s001.doc]

| Genus | Species | Mass (kg) | Humerus Length (mm) | Humerus Cranio-Caudal Breadth | Humerus Dorso-Ventral Breadth | Femur Length | Femur Medio-Lateral Breadth | Femur Dorso-Ventral Breadth | Humerus Zx (solid) | Humerus  Zy (solid) | Humerus Zy/Zx | Femur Zx (solid) | Femur Zy (solid) | Zy Ratio (hum/fem) | Femoral strength (Femur Zp/Mass) | Humeral strength  (Humerus Zp/Mass) | Femur Zp/Mass |
| --- | --- | --- | --- | --- | --- | --- | --- | --- | --- | --- | --- | --- | --- | --- | --- | --- | --- |
| *Pygoscelis* | *papua* | 4.80 | 80.22 | 16.83 | 6.02 | 89.09 | 9.01 | 9.14 | 35.85 | 280.60 | 7.83 | 60.57 | 58.82 | 4.77 | 24.87 | 65.93 | 24.87 |
| *Pygoscelis* | *adeliae* | 3.94 | 68.79 | 14.42 | 5.24 | 79.33 | 7.99 | 8.46 | 23.69 | 179.38 | 7.57 | 47.82 | 42.63 | 4.21 | 22.95 | 51.53 | 22.95 |
| *Eudyptes* | *chrysocome* | 2.19 | 57.99 | 11.31 | 4.35 | 67.96 | 6.77 | 7.46 | 12.56 | 85.02 | 6.77 | 32.49 | 26.70 | 3.26 | 27.09 | 44.66 | 27.09 |
| *Eudyptula* | *minor* | 0.90 | 45.12 | 7.76 | 3.36 | 52.94 | 4.99 | 5.13 | 5.13 | 27.35 | 5.33 | 9.97 | 9.46 | 2.89 | 21.59 | 36.09 | 21.59 |
| *Aptenodytes* | *patagonica* | 16.00 | 105.43 | 20.74 | 8.13 | 97.22 | 11.47 | 13.38 | 83.01 | 540.07 | 6.51 | 221.86 | 163.22 | 3.31 | 24.07 | 38.94 | 24.07 |
| *Aptenodytes* | *forsteri* | 29.50 | 123.19 | 24.31 | 10.38 | 119.65 | 15.24 | 17.19 | 173.34 | 950.77 | 5.48 | 507.98 | 399.01 | 2.38 | 30.75 | 38.11 | 30.75 |
| *Spheniscus* | *demersus* | 3.04 | 66.32 | 11.87 | 5.02 | 76.60 | 7.80 | 7.99 | 17.72 | 99.20 | 5.60 | 40.72 | 38.86 | 2.55 | 26.20 | 38.50 | 26.20 |
| *Cerorhinca* | *monocerata* | 0.57 | 64.80 | 5.27 | 3.60 | 37.88 | 2.62 | 3.54 | 2.98 | 6.39 | 2.14 | 2.41 | 1.32 | 4.84 | 6.51 | 16.35 | 6.51 |
| *Fratercula* | *arctica* | 0.48 | 61.94 | 4.87 | 3.35 | 38.37 | 2.93 | 3.13 | 2.31 | 4.88 | 2.12 | 1.84 | 1.61 | 3.03 | 7.19 | 14.99 | 7.19 |
| *Alca* | *torda* | 0.66 | 81.26 | 7.31 | 3.76 | 44.26 | 3.84 | 4.35 | 3.74 | 14.15 | 3.78 | 5.60 | 4.35 | 3.25 | 15.04 | 27.02 | 15.04 |
| *Uria* | *aalge* | 1.01 | 87.36 | 8.35 | 4.01 | 48.63 | 4.01 | 4.76 | 4.84 | 20.99 | 4.34 | 6.98 | 4.96 | 4.23 | 11.87 | 25.68 | 11.87 |
| *Tonsala* | *buchanani* | 30.88 | 149.70 | 20.51 | 9.23 | 141.00 | 14.71 | 16.68 | 84.60 | 417.78 | 4.94 | 380.25 | 295.74 | 1.41 | 21.89 | 16.27 | 21.89 |
| *Tonsala* | *hildegardae* | unknown | unknown | unknown | unknown | 105.38 | 10.15 | 11.91 | unknown | unknown | unknown | 127.80 | 92.82 | unknown |  |  |  |
| *Tonsala* | *hildegardae* | unknown | unknown | unknown | unknown | 105.38 | 10.40 | 11.83 | unknown | unknown | unknown | 128.33 | 99.18 | unknown |  |  |  |
